# LC/MS/MS data analysis of the human uterine smooth muscle S-nitrosoproteome fingerprint in pregnancy, labor, and preterm labor

**DOI:** 10.1016/j.dib.2015.07.031

**Published:** 2015-08-01

**Authors:** Craig C. Ulrich, David R. Quilici, Karen A. Schlauch, Heather R. Burkin, Iain L.O. Buxton

**Affiliations:** a University of Nevada School of Medicine, Department of Pharmacology, United States; b Nevada Proteomics Center, Reno, Nevada, United States; c University of Nevada, Reno, Department of Biochemistry and Molecular Biology, United States

## Abstract

The data described in this article is the subject of an article in the American Journal of Physiology: Cell Physiology, titled “The Human Uterine Smooth Muscle S-nitrosoproteome Fingerprint in Pregnancy, Labor, and Preterm Labor” (doi:10.1152/ajpcell.00198.2013) (Ulrich et al., 2013) [1]. The data described is a large scale mass spectrometry data set that defines the human uterine smooth muscle S-nitrosoproteome differences among laboring, non-laboring, preterm laboring tissue after treatment with S-nitrosoglutathione.

Specifications Table

**Table:** 

Subject area	*Pharmacology*
More specific subject area	*Reproductive Biology, Preterm Labor*
Type of data	*.XML and. RAW files*
How data was acquired	*Mass Spectrometry (Thermo-Fisher LTQ-Orbitrap)*
Data format	*Filtered XML files and RAW files*
Experimental factors	*Samples were treated with S-nitrosoglutathione and S-nitrosated proteins were isolated by the biotin-switch technique.*
Experimental features	*LC/MS/MS analysis of enriched S-nitrosated proteins from human uterine smooth muscle in disparate states of pregnancy.*
Data source location	*PRIDE Archive*
Data accessibility	*Freely available in the PRIDE Archive (Project PXD000226)**http://www.ebi.ac.uk/pride/archive/projects/PXD000226*

**Value of the data**

This is the first and only description of nitric oxide (NO)-mediated post-translational modification in human uterine smooth muscle.

• This data provides a target list for future work delineating nitric oxide signaling pathways in human pregnancy.
• Because relaxation of the uterine smooth muscle is independent of soluble guanylyl cyclase activation and cGMP accumulation, this data set likely includes the S-nitroso proteins responsible for NO-mediated relaxation.
• The raw data can be used by all smooth muscle and reproductive scientists to formulate their own unique hypotheses regarding the role of nitric oxide in the posttranslational modification of cysteine residues in smooth muscle.

## 1 Data, experimental design, materials and methods

### 1.1 Data

The data in the PRIDE Archive provide a novel description of the NO-induced posttranslational modifications that were identified in human uterine smooth muscle during disparate states of pregnancy. This data can be analyzed using a variety of free and commercial tandem mass spectrometry tools currently available, such as MaxQuant and the Trans-Proteomic Pipeline. We have provided a descriptive MS/MS data set of peptides identified in human uterine smooth muscle that show disparate reactivity to S-nitrosoglutathione in different pregnancy states. The following materials and methods, as well as data parsing sections, will enable investigators to design novel procedures involved in NO signaling that rely on MS/MS techniques.

## 2 Materials and methods

### 2.1 Chemicals

Sodium ascorbate, *N*-2-hydroxyethylpiperazine-*N*′-2-ethanesulfonic acid (HEPES), neocuproine,*N*-ethylmaleimide (NEM), methyl methanethiosulfonate (MMTS), 3-(3-cholamidopropyl)dimethylammonio-1-propanesulfonate (CHAPS), sodium dodecyl sulfate (SDS), and all other chemicals unless specified were obtained from Sigma (St. Louis, MO). *N*-[6-(biotinamido)hexyl]-3′-(2′-pyridyldithio) propionamide (biotin-HPDP) was purchased from Thermo Scientific (Rockford, IL).

### 2.2 Tissue collection

All research works were reviewed and approved by the University of Nevada Biomedical Review Committee for the protection of human subjects. Human uterine myometrial biopsies were obtained with written informed consent from mothers undergoing elective Cesarean section in preterm labor without infection or premature rupture of membranes; term in labor or term not in labor. Tissues were transported to the laboratory immediately in cold physiological buffer, microdissected under magnification to isolate smooth muscle, employed in contractile experiments or snap frozen in liquid nitrogen, and stored at −80 °C. The average age for patients in the pregnant laboring group was 28.9±5.6 yr in the nonlaboring group 28±5.1 yr and in the preterm laboring group 30.8±10.2 yr. Pregnant laboring and nonlaboring patients ranged from 37 to 41 wk gestation, with the mean at 39 wk for both laboring and nonlaboring groups. Preterm laboring patients ranged from 29.2 to 36 wk of gestation, with the mean being 33.5 wk. Patients represented a range of ethnicities and were 52% Caucasian, 30% Hispanic, 7.4% African American, and 11% other.

### 2.3 Protein isolation

To isolate total protein, myometrial muscle samples from 12 patients in each pregnancy state were ground to a powder under liquid nitrogen and reconstituted in 20 ml HEN buffer (25 mM HEPES-NaOH, 1 mM EDTA, and 0.1 mM neocuproine, pH 7.7). Samples were sonicated (10×2- s bursts, 70% duty cycle) and brought to 0.4% CHAPS. Samples were then centrifuged at 2000*g* for 10 min at 4 °C. Protein concentration was determined by the bicinchoninic acid assay and samples diluted to 0.8 mg/ml in HEN buffer.

### 2.4 Biotin switch and streptavidin pull-down

Samples from each patient in all groups were independently isolated by biotin switch and streptavidin pulldown and then pooled for tandem mass spectrometry (MS/MS) analysis (i.e., PTL1, 4 unique patients; PTL2, 4 unique patients; PTL3, 4 unique patients; for a total of 12 unique patients split into 3 biological replicates to help control for human diversity). Unless stated, all steps of the biotin switch were performed in the dark. Protein isolates (1.8 ml 0.8 mg/ml in HEN buffer) were incubated with GSNO (1746 μl of sample+54 μl of 10 mM GSNO prepared in the dark) for 20 min at room temperature. We selected a concentration for this labeling of 300 μM because at this concentration, GSNO will produce ~5 μM reactive NO over 15–20 min without accumulation [2]. This reactive species concentration matches the IC_50_ concentration for relaxation of isolated myometrium [3]. Noncysteinyl nitrosation events would not be appreciated due to the reactive chemistry of ascorbate reduction; a nucleophilic attack at the nitroso-nitrogen atom leading to thiol and *O*-nitrosoascorbate (*reaction 1*)

RSNO+ASC→RSH+NOASC

which breaks down, by various competitive pathways, the dominant of which at physiological pH yields dehydroascorbic acid and nitroxyl radical that decomposes at physiological pH to nitrous oxide [4] (*reaction 2*).

NOASC+H^+^→DHA+HNO

2HNO→HON=NOH

HON=NOH→N_2_O+H_2_O

Neither biotin-HPDP nor a maleimide dye would lead to false positives because the amines or tyrosines would not be labeled even if they were nitrosated. SDS (0.2 ml of 25% SDS in water) was added along with 20 μl of 3 M NEM (final 2.0 ml at 2.5% SDS and 30 mM NEM). Samples were incubated at 50 °C in the dark for 20 min with frequent vortexing. Three volumes of cold acetone (6 ml) were added to each sample. Proteins were precipitated for 1 h at −20 °C and collected by centrifugation at 3000*g* for 10 min. The clear supernatant was aspirated, and the protein pellet was gently washed with 70% acetone (4×5 ml). After resuspension in 0.24 ml HEN buffer with 1% SDS (HENS), the material was transferred to a fresh 1.7- ml microfuge tube containing 30 μl biotin-HPDP (2.5 mg/ml). The labeling reaction was initiated by addition 30 μl of 200 mM sodium ascorbate (final 20 mM ascorbate) for 1 h at room temperature in the dark. Four volumes of −20 °C acetone were added to the labeled samples and incubated at −20 °C for 20 min to remove biotin-HPDP. The samples were centrifuged at 3000*g* for 10 min at 4 °C, and the supernatant was discarded. The sides of the tube and the pellet were washed with −20 °C acetone to remove traces of biotin-HPDP. The pellet was resuspended in 140 μl of HENS buffer. Neutralization buffer (20 mM HEPES pH 7.7, 100 mM NaCl, 1 mM EDTA, and 0.5% Triton X-100) was added (280 μl) along with 42 μl of streptavidin-agarose. Proteins were incubated for 1 h at room temperature and washed five times with 1.5 ml of neutralization buffer with 600 mM NaCl. Beads were incubated with 100 μl elution buffer (neutralization buffer with 600 mM NaCl plus 100 mM β-mercaptoethanol) to recover the bound proteins. This step releases the protein from the streptavidin bead leaving the biotin-HPDP tag bound to the bead as well as natively biotinylated proteins still bound to the bead. Four volumes of −20 °C acetone were added, and samples were incubated for 1 h at −20 °C to reprecipitate proteins. Samples were centrifuged at 3000*g* for 10 min at 4 °C, the supernatant was discarded, and the pellet was washed and dried for proteomic analysis.

### 2.5 Protein digestion and mass spectrometry

The Nevada Proteomics Center analyzed selected proteins by trypsin digestion and liquid chromotography (LC)/MS/MS analysis. Acetone-precipitated pellets were washed twice with 25 mM ammonium bicarbonate and 100% acetonitrile, reduced, and alkylated using 10 mM dithiothreitol and 100 mM iodoacetamide and incubated with 75 ng sequencing grade-modified porcine trypsin (Promega, Fitchburg WI) in 25 mM ammonium bicarbonate overnight at 37 °C. Peptides were first separated by Michrom Paradigm Multi-Dimensional Liquid Chromatography (MDLC) instrument [Magic C_18_AQ 3μ 200 Å (0.2×50 mm) column (Michrom Bioresources, Auburn, CA) with an Agilent ZORBAX 300SB-C_18_ 5μ (5×0.3 mm) trap (Agilent Technologies, Santa Clara, CA)]. The gradient employed 0.1% formic acid in water (*pump A*) and 0.1% formic acid in acetonitrile (*pump B*) as follows time (min), flow (μl/min), *pump B* (%): (0.00, 4.00, 5.00), (5.00, 4.00, 5.00), (95.00, 4.00, 45.00), (95.10, 4.00, 80.00), (96.10, 4.00, 80.00), (96.20, 4.00, 5.00). Eluted peptides were analyzed using a Thermo Finnigan LTQ-Orbitrap using Xcalibur v 2.0.7. MS spectra (*m*/*z* 300–2000) were acquired in the positive ion mode with resolution of 60,000 in profile mode. The top 4 data-dependent signals were analyzed by MS/MS with CID activation, minimum signal of 50,000, isolation width of 3.0, and normalized collision energy of 35.0. The reject mass list included the following: 323.2040, 356.0690, 371.1010, 372.1000, 373.0980, 445.1200, 523.2840, 536.1650, 571.5509, 572.5680, 575.5494, 677.6090, 737.7063, 747.3510, 761.7316, 763.8791, 767.0623, 824.4870, 832.1884, 930.1760, 1106.0552, 1106.0564, 1142.0940, and 1150.0927. Dynamic exclusion settings were used with a repeat count of two, repeat duration of 10 s, exclusion list size of 500, and exclusion duration of 30 s.

### 2.6 Data processing and upload to the PRIDE repository

All raw data acquired on the LTQ-Orbitrap is available in the PRIDE Repository in the Thermo Fisher.RAW format. All.RAW,.MS2, and.MGF data was translated into the PRIDE XML format and is freely available in the PRIDE repository at the following web address: http://www.ebi.ac.uk/pride/archive/projects/PXD000226.
